# Polydopamine Films with 2D-like Layered Structure and High Mechanical Resilience

**DOI:** 10.1021/acsami.1c02483

**Published:** 2021-05-10

**Authors:** Emerson Coy, Igor Iatsunskyi, Juan Carlos Colmenares, Yeonho Kim, Radosław Mrówczyński

**Affiliations:** † NanoBioMedical Centre, Adam Mickiewicz University, Wszechnicy Piastowskiej 3, 61-614 Poznan, Poland; ‡ Institute of Physical Chemistry, Polish Academy of Sciences, Kasprzaka 44/52, 01-224 Warsaw, Poland; § Research Institute of Basic Sciences, Incheon National University, Incheon 22012, Republic of Korea; ∥ Faculty of Chemistry, Adam Mickiewicz University, ul. Uniwersytet Poznańskiego 8, 61-614 Poznań, Poland

**Keywords:** polydopamine, layered structures, air−water interface, nanoindentation, protomolecules

## Abstract

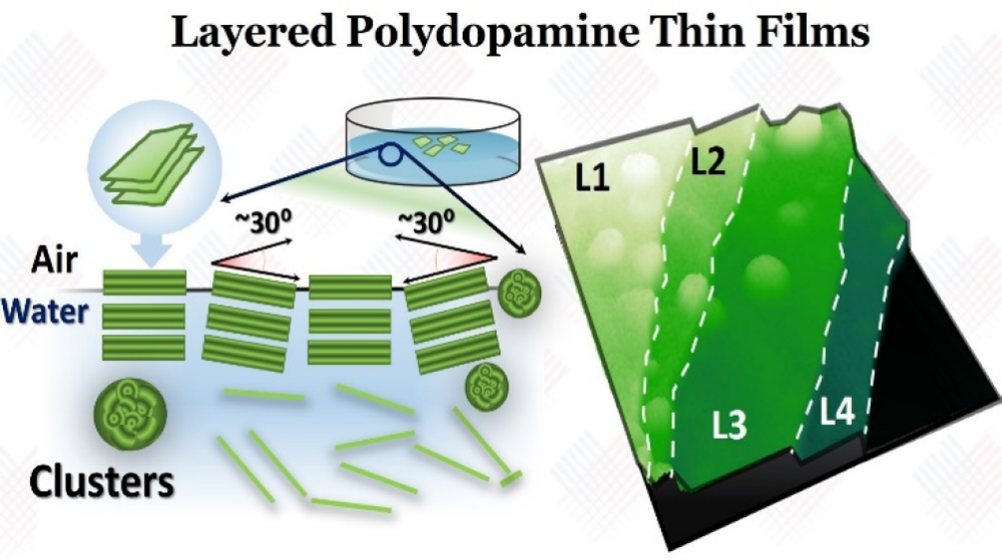

Highly oriented, layered, and mechanically resilient films of polydopamine (PDA) have been synthesized from the air/water interface. The films show a unique layered structure, as shown by scanning and transmission electron studies (SEM/TEM) and X-ray diffraction analysis (XRD), which resemble that of 2D layered materials. The films exhibit a composition typical of PDA-based materials, as evidenced by X-ray photoelectron spectroscopy (XPS); moreover, the samples present the distinctive resonance modes of PDA-based nanomaterials in Raman and infrared spectroscopy (FTIR) experiments. The presence of highly ordinated 3–4 protomolecule stacking, taking place at the air/water interface, with a unique eumelanin-like supramolecular arrangement is presented. Moreover, the films show superior mechanical resilience with *E* = 13 ± 4 GPa and *H* = 0.21 ± 0.03 GPa, as revealed by nanoindentation experiments, making them highly resilient and easily transferable. Finally, the ordering induced by the interface opens many possibilities for further studies, including those regarding the supramolecular structure on PDA due to their similarity to 2D layered materials.

## 1 Introduction

The mussel-inspired polydopamine (PDA) coating has found a widespread application in preparation materials, thanks to its relatively simple polymerization pathway,^1−3^ resulting in a broad spectrum of fields, ranging from chromatic control^4−6^ to antibacterial applications,^7^ and more recently in fields such as organic/metal/photocatalysis and energy production.^8−13^ Additionally, an important area for polydopamine application is in biomedicine,^14^ where it has shown compelling results in drug delivery and tissue engineering^15−17^ due to its biocompatibility and straightforward synthesis.^18−20^ With regard to the synthesis of PDA materials, the vast majority of these composites are obtained by the oxidative polymerization of dopamine in an alkaline buffer in the presence of substrate.^20,21^ The dopamine polymerization process can be controlled by tuning the nature of the oxidant, temperature, and time of reaction as well as by radical scavenger selection.^22,23^

The majority of the applications of PDA and studies attempt to understand the physicochemical characteristics of PDA layers grown on solid supports or directly address amorphous PDA particulates obtained during polymerization.^1,2,24,25^ However, over the past few years, some studies have explored the possibility of obtaining thin PDA membranes synthesized at the water/air interphase.^26−31^ The general attractiveness of this methodology relies on the larger area of PDA films, homogeneity, and easily transferability. Despite their applicability and the rather exceptional properties of PDA, a few studies have shown the use of PDA membranes as wound dressing tools^32^ and as a calcination template for the oxygen reduction reaction.^33^ However, the structure of these PDA layers is typically amorphous and does not show any self- or supramolecular organization, despite the similarities between PDA with the eumelanin protomolecule stacking configuration.^34,35^ Nevertheless, the stacking formation should, in principle, allow for the presence of a pseudo-2D organization in PDA membranes, with little or no long-range ordering.

In this work, we describe the synthesis and characterization of such PDA membranes obtained from dopamine polymerization in Tris buffer and their self-assembly in pseudolayered structures. We investigate their structure, optical properties, and microscopic features as well as their mechanical response after transferring to commercially available silicon substrates. The results here presented show a previously unreported, highly oriented layered structure with clear similarities to graphite/graphene oxide materials. We also evaluate the superior mechanical response by nanoindentation, with a Young’s modulus (*E*) of 13 ± 4 GPa and hardness (*H*) of 0.21 ± 0.03 GPa, which allows for the easily transferability of the film to other substrates and even relative large free-standing architectures. Additionally, we propose a mechanism for the formation of these layers using a combination of the previously described eumelanin protomolecule stacking and the air/water interface.

## 2 Experimental Methods

### Synthesis of Polydopamine Membranes

The synthesis of PDA membranes was performed in a Petri dish (14 cm in diameter) containing Tris buffer solution (pH = 8.5, 10 mmol, 40 mL) followed by the addition of dopamine (2 mg mL^–1^). The small stirring bar was put in the Petri dish and rotated at 300 rpm. Finally, the vessel was covered with a glass lid. For thick samples, a piece of silicon was immersed in the Petri dish, with a corner covered by Kapton tape. A conventional lime soda glass microscope slide was also placed in the bottom of the dish for control. The polymerization process was conducted for 24 h with a nonsealed cover lid (a larger Petri dish) under flowing air from the fume hood. Then the lid was taken out to scoop the thin layers floating on the solution’s surface by using commercially available Si(001) substrates with native SiOx and mica glasses. The rest of the reaction was left under the hood until the solution dried, producing the thick layers on the exposed areas of the Si and microscope slide substrates.

### Characterization of the Samples

We characterized samples by X-ray diffraction (XRD) and grazing incident X-ray diffraction (GiXRD) using an MRD-X’pert^3^ diffractometer (PANalytical), working in 45 kV and 40 mA (Cu source). The out-of-plane ordering of the samples was examined by performing GiXRD scans at different Ψ angles (0–45°). Scanning electron microscopy (SEM) images were collected in a JEOL 7001TTLS (JEOL) working at 1 kV, without sputtering cover layers. Atomic force micrographs (AFM) were collected in an ICON (Bruker) microscope working in tapping mode. High-resolution transmission electron images (HR-TEM) were collected in an ARM-200f (JEOL) microscope, working at 80 kV and equipped with an EDX detector. Commercially available bare copper grids (100 mesh) with ∼204 μm hole width (Ted Pella) were used to manually scoop the PDA membranes. Samples were left to dry overnight in ambient conditions. Infrared spectroscopy (FT-IR) was performed by using an FT/IR-4700 (Jasco). Raman studies were performed by using a 633 nm laser, in backscattering geometry in an NT-MDT (Renishaw). Finally, nanoindentation experiments were performed by using a TI-950 (Hysitron) triboindenter equipped with a Berkovich tip. Load and displacement curves were analyzed according to the Oliver and Pharr method^36,37^ and methodology described elsewhere.^38^

## 3 Results and Discussion

### Physicochemical Characterization

**Figure 1:**
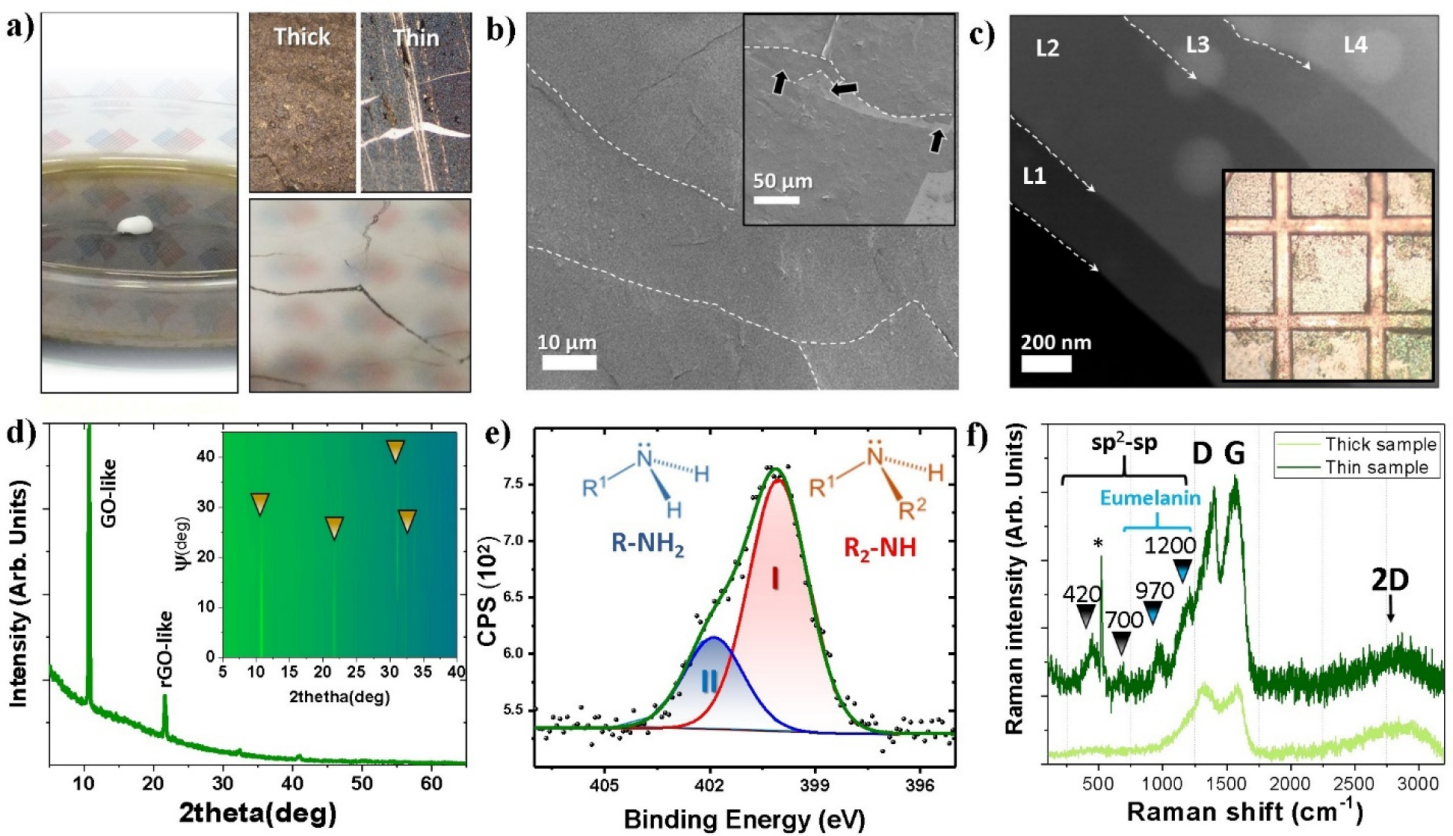
(a) (left) Image of the synthesis dish used for the PDA polymerization; (bottom right) image of the reflective surface of the Petri dish with a membrane connecting and breaking after the scooping by silicon. (top right) Optical image of the scooped and thick layers of PDA on polished silicon. (b) SEM images of the surface of thin PDA samples at different magnifications. Dashed lines show grain boundaries and black arrows the layered structure. (c) STEM micrographs of transferred sample on TEM grids. L1 to L4 show the increment of layers found in the samples; dashed lines show the borders. Inset shows an image of the grid under a microscope (grid size 204 μm). (d) XRD diffractogram for thick PDA sample. Inset shows the Ψ vs 2Θ–ω scan with yellow arrows pointing to diffraction peaks. (e) Shows two main nitrogen (N 1s) species observed in the thin sample by XPS. (f) Raman spectra for both thin and thick samples marking the D, G, and 2D distinctive peaks. Additionally, the black arrows show the additional vibration observed for the thin sample. (∗) points to the silicon substrate vibration. The cyan region shows the vibration that resembles those reported for eumelanin.

As described in the Experimental Methods section, two main PDA samples were prepared in this study, a thick and a thin sample; images of the samples are shown in Figure S1. The thick sample (i) refers to a silicon piece placed at the bottom of the solution, which collected several layers of PDA after the evaporation of the solution. The thin sample (ii) refers to the scooped membrane from the surface of the dish after 24 h of synthesis. The synthesis vessel is presented in Figure 1a, showing the Petri dish and the air/water interface at the surface of the synthesis solution after 24 h. Figure 1a inset shows a microscope image of the thick and thin samples. Figure 1b shows the SEM micrographs obtained for the thin samples, showing what resembles large grain boundaries (marked with dashed lines in the image) and what resembles an overlapping layered structure (Figure 1b, inset) marked with arrows. Figure 1c shows the STEM micrographs of a free-standing section of the thin sample scooped on a Cu grid. A clear layered section is visible in the image, identified as L1 to L4 in the image caption. The layers’ borders are marked by dashed lines. It is important to notice that along with the layered structure some spherical PDA particles are found on the membranes (Figure S2a). Further inspection using selected area electron diffraction (SAED) patterns show no in-plane discernible organization, and HR-TEM micrographs show no distinguishable structures in the nanometers range. Additional STEM images and SAED patterns are shown in Figure S2b. The elemental distribution and quantification were performed by EDX; the calculation was made by analyzing five independent measurements in different regions of the sample (Figure S2c). The pondered results show mainly C(80.9%), O(3.6%%), and N(15.4%) signals, clearly localized on the membrane section of the sample. Electron energy loss spectroscopy (EELS), aiming to identify the thickness of the layers (zero loss), shows an overage thickness of ∼55 nm per membrane layer (Figure S3). At this point, it is suspected that the observed layered structures belong to a sort of PDA supramolecular structure or a layered ordering of the PDA membrane, which has not been observed before.

Figure 1d shows the diffractogram collected for the thick sample. The diffractogram for the thin sample shows similar features to that of the thick sample (Figure S4). The diffractogram shows a set of peaks at ∼10.7° (*d* = 8.3 Å), ∼21.6° (*d* = 4.1 Å), ∼32.4° (*d* = 2.8 Å), and ∼41° (*d* = 2.2 Å), with reducing in intensity at higher 2Θ angles. Grain sizes calculated by the Scherrer equation are ∼10.7° (69.5 nm), ∼21.6° (37.6 nm), and ∼32.4° (12.9 nm) after geometrical adjustments.^39^ The values are smaller than the observed membranes and contrast with the lack of visible grain boundaries on SEM and TEM images. As it is well documented in the literature, PDA is an amorphous polymer that does not show any distinctive crystallographic features. Therefore, the presence of these peaks needs to arise from other structural order. It is striking the resemblance of the observed XRD peaks and their interplanar distances with those of well-known carbon materials, such as graphene oxide (GO), ∼10° and ∼40°,^40^ reduced graphene oxide (rGO), ∼20°,^41^ and other carbon materials.^42^ Despite their similarities, it is clear that there is no synthesis pathway between dopamine and Tris buffer, capable of resulting in 2D-graphene-based materials; thus, the observed structure must be a PDA analogue of a 2D-carbon material. A useful method to determine if the 2D-like domains are perpendicularly aligned with the substrate, or randomly oriented within the film, is the 2Θ scan at different Ψ angles as shown in the Figure 1d inset. As the Ψ increases, the intensity of the peaks decreases and disappears at Ψ ≈ 30°; this behavior shows that the van der Waals planes are predominantly parallel to the substrate interface and therefore the air/water interface.

Chemical studies performed by XPS on the thin sample are shown in Figure 1e (Figure S5 for other components and Table S1 for quantification). The high-resolution N 1s region collected for the thin sample shows the presence of two distinctive nitrogen species centered at 400 eV (3.05% at conc) and 401.9 eV (1.11% at conc), which can be assigned to R_2_–NH and R–NH_2_, respectively, moieties present in PDA materials.^18,43^ One of the most well-established techniques for investigating both van der Waals and carbon materials is Raman spectroscopy.^44^ It is a referent for both 2D and pseudo-2D materials as it reflects allows the in-detail investigation of their structure. Figure 1f shows Raman spectra collected for both thin and thick samples. Both spectra show the clear presence of the D (1200–1429 cm^–1^) and G (1500–1630 cm^–1^) peaks, typical for carbonaceous materials and commonly observed in PDA materials;^45^ moreover, there is a small but clear signature for the second-order process by the apparition of the *G*′, also known as the 2D (2600–3000 cm^–1^) peak.^46^ A comparison between the *I*_D_/*I*_G_ ratio for thick and thin samples shows a ratio = 0.83 and 0.88, respectively, which are surprisingly low. Such low values are typically found in pyrolyzed or graphitized PDA,^47−49^ which is surprising since there is no chemical route for graphitization in our preparation. Nevertheless, applying the generalized equation for crystalline size (*L*_a_),^50^ we obtain a *L*_a_ = 42.5 nm. This value, although slightly inferior to the ones measured by XRD, should contain only information about the pseudo-graphitic (π–π) components of the layers instead of purely graphitic ones. More importantly, the thin sample shows a group of unreported minor signals, not commonly observed for a PDA-based material. Some of the vibrations can be understood by their resemblance to the experimental and theoretical results for eumelanin, specifically the 940–1045 cm^–1^ vibration, attributed to the C–H, O–H, and O–H deformations, and the 1100–1242 cm^–1^ vibration, attributed to the stretching of C–C, C–OH, C–H, and N–H bound present in amide, phenolic, and pyrrole groups.^51^ The lower frequencies at 700 and 420 cm^–1^ are difficult to assign to any specific organic compound; however, these two bands are often observed in defective and twisted carbons, which have been proposed as an indication of the less presence of sp^2^ in carbonaceous samples, especially the ∼400 cm^–1^ band^52,53^ previously observed in the literature.^54^ Additionally, the band at 700 cm^–1^ is attributed to the sp^2^ amorphous phase activated by cooperative disorder in the sample,^55^ which is a feature that melanin shares with partially disordered carbon materials.^34^ Furthermore, these studies have also shown some overlapping with the bands attributed to eumelanin and those of carbon materials, which complicates the distinction between these contributions.

**Figure 2:**
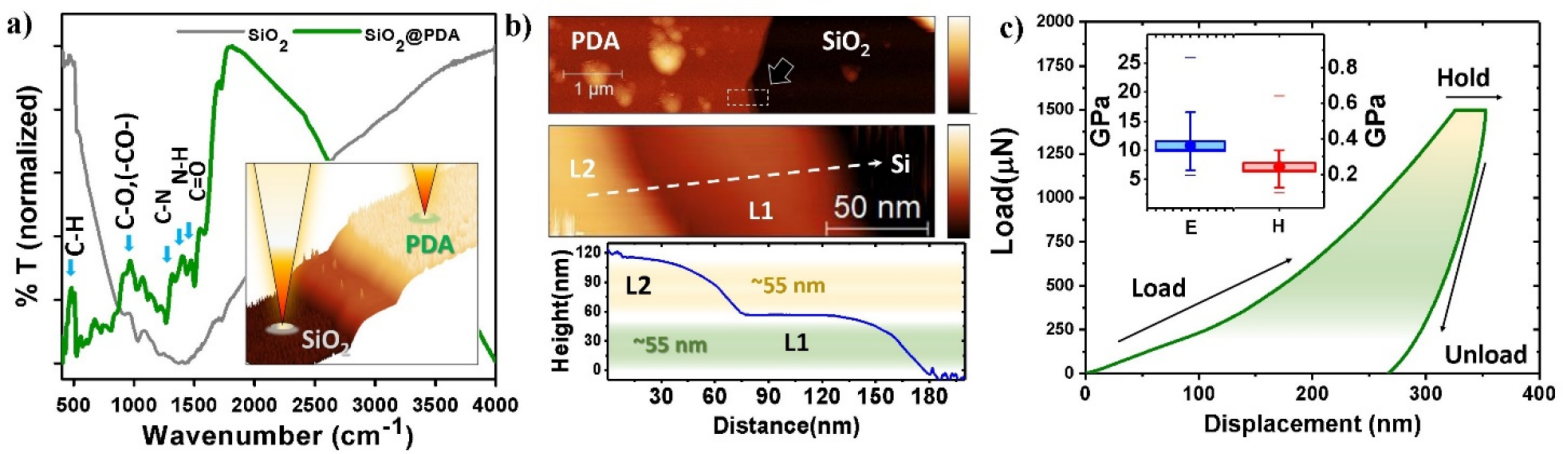
(a) Infrared transmission spectroscopy studies of the thick membrane samples and silicon substrate. Arrows point to the specific vibration observed in PDA. The inset shows the inspected regions. (b) Atomic force micrographs collected from the thin sample on silicon. The top panel shows a large-scale image of one of the boundary sections of the PDA film. Dashed square and black arrows show a section in which the presence of a double layer is observed. The middle panel shows a zoomed-in micrograph of this section and the presence of three regions, marked as L1, L2, and silicon. The dashed arrow shows the profile of the image, which is represented in the bottom panel of the same image. (c) A representative load vs displacement curve obtained from the thick sample. The inset shows the box distribution of the *E* and *H* for the sample.

FTIR studies aiming to address the chemical structures present in the samples are shown in Figure 2a. The spectrum of the PDA thick layer exhibits peaks in four different areas. The silicon substrate is also presented for comparison. The broad band between 3600 and 3100 cm^–1^ with a peak at 3369 cm^–1^ is assigned to stretching modes of ν(N–H) and ν(O–H) from amino and hydroxyl moieties. The peaks at 2969, 2922, and 2847 cm^–1^ correspond to stretching C–C modes from aliphatic CH_2_ groups present in polydopamine. The peak at 1726 cm^–1^ is due to the presence of carbonyl ν(C=O) from quinones. The signals in the range between 1591 and 1340 cm^–1^ result from stretching modes of ν_ring_(C=C) and ν_ring_(C=N), 1460 cm^–1^ ν_ring_(C=C), and 1354 cm^–1^ ν_ring_(CNC) from aromatic amines and the indole ring, clearly proving the PDA layer on the support. Those data are in agreement with previously published spectra of PDA layers.^56^ However, it is impossible to conclude more detailed structural properties of polydopamine by using the FTIR technique.

Nevertheless, the distinctive structural differences (XRD/SEM/TEM) of the membranes here presented, when compared with other PDA films,^54,57^ show a promising panorama for further studies.

### Nanomechanical Response

One important aspect to study is the superior mechanical strength shown by the PDA thin films. This is first intuitively observed in the rather large suspended area on the TEM grid (Figure 1c). Previously, the mechanical response of the PDA films was evaluated by nanoindentation.^58^ The authors reported an *E* = 2.3 ± 0.84 GPa, which increased to >14 GPa upon calcination (600 °C). The films were prepared in a somehow similar method; however, pristine membranes were subjected to a heat treatment (300 °C) before mechanical analysis, which resulted in cracks and shrinkage of the membranes. More recent studies have shown free-standing membranes of PDA synthesized by electrochemical methods.^52^ The mechanical properties of these films were investigated by micro-Brillouin light spectroscopy.^59^ The authors reported a much higher Young’s modulus of ∼12 GPa, which allowed the easy transfer of the films.

To investigate the nanomechanical response of the PDA layers, we first collected atomic force micrographs of the samples, as shown in Figure 2b. As previously measured by EELS, the individual layers of PDA show a thickness of 55 nm with an overall roughness of *R*_q_ = 2 nm for the thin sample. We performed a series of indentation tests on the thick PDA sample surface (Figure 2c); the penetration depth was kept at 320 nm at a 1500 μN load. To collect enough statistical information, a sampling population of around 100 indents was collected. Additionally, shallow indents with a maximum penetration of 48 nm and a load of 50 μN were also performed; similar studies were performed for the thin sample. As shown in the inset of Figure S6, however, the influence of the substrate prevented the accurate calculation. The results show a general value of *E* = 13 ± 4 GPa and *H* = 0.21 ± 0.03 GPa for the PDA samples. The recorded mechanical response should in practice resemble that of bulk PDA, which given the magnitude of the *E* and *H* explains the rather large mechanical stability of the PDA films, similar to the one reported in the literature.^59^

### Apparition of the Layered Structure in PDA

The physicochemical properties of PDA show significant similarity to those of naturally occurring eumelanin. Thus, the conversion of dopamine to PDA is explained by applying the Raper–Manson mechanism, which is frequently used to explain the formation of eumelanin.^60,61^ According to this mechanism, dopamine is oxidized to dopamine quinone, which later cyclizes to lecodopaminechrome. In addition, isomerization and oxidation of lecodopaminechrome lead to 5,6-dihydroxyindole, which is considered as the main monomer of PDA. The cross-linkage of the 5,6-dihydroxyindole at the positions 4, 7, and 2 results in the typical heterogeneous, black, and insoluble material we know as PDA. Up to now, different structural models of PDA have been reported^62−65^ (Figure S7). Nevertheless, the PDA structure remains elusive, and it is still under debate in the literature.

**Figure 3:**
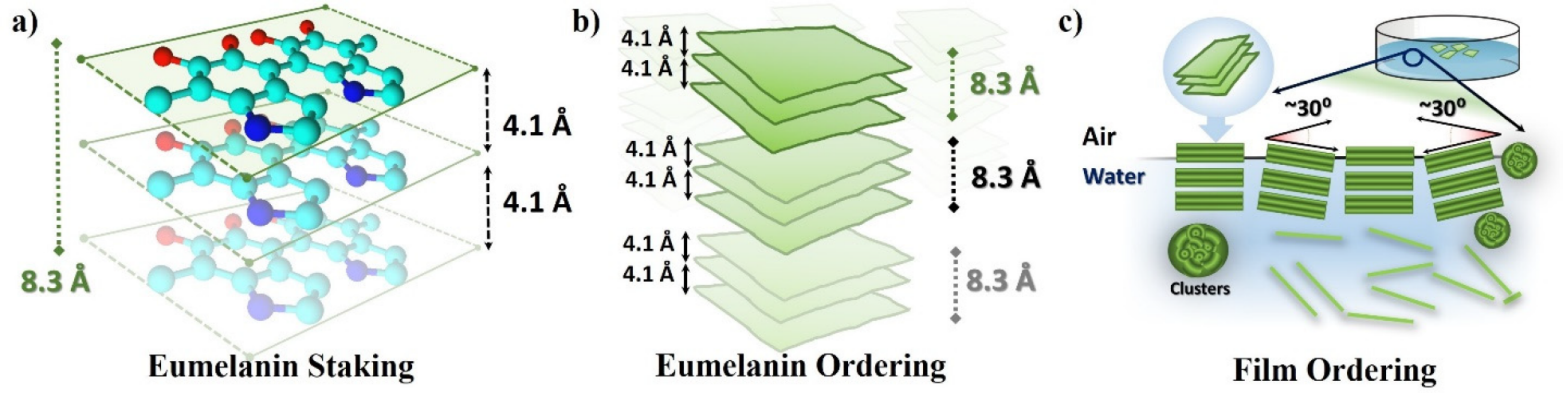
(a) Representation of eumelanin molecule in 3D. Red atoms are oxygen, while blue are nitrogen. The representation shows the stacking of the molecules, with the interplanar distance observed in this study. Also, the high of the triple-stacked eumelanin monomer is shown with an estimated value of 8.3 Å, which is compatible with three stacked molecules. (b) Proposed eumelanin superior order, where a few groups of three stacked protomolecules are ordered with an 8.1 Å periodicity. (c) Air/water interface showing the proposed ordering of the membranes and the protomolecule clusters seeding on the membranes. The angle of ∼30° is marked as measured by X-ray diffraction Ψ-scans.

Based on the similarity of PDA to eumelanin, there are a few options for the PDA structure observed in this study.^66^ First, the protomolecule stacking has been proposed and investigated extensively to show the stacking of 3–4 layers consisting of five to seven monomers of indole-5,6-quinone. The interplanar distance of this stacking has been extensively studied from naturally occurring and synthetic sources, and it is reported as 3.4–4.7 Å,^34,67−73^ which properly correspond to the second register peak in Figure 1d, ∼21.6° (*d* = 4.1 Å), and are represented in Figure 3a. Moreover, other supramolecular arrangements have been observed before, such as a 7–8 Å peak^67^ observed for natural eumelanin, which is typically explained by the partially destacking of the population.^34^ More interestingly, this value also suits the experimental height of the 3–4 protomolecule stacking (7.4 Å)^70^ and is somehow close to the ∼10.7° (*d* = 8.3 Å) peak observed in the samples, which could suggest a superior ordering and periodic organization of 3–4 stacked protomolecules in the film, as presented in Figure 3b. One additional aspect to consider is the presence of nanoaggregates, which share some similarities with the structures observed in this study^72,74^—more specifically the nanoparticles accompanying the membranes (Figure 1c), in which both the ∼32.4° (*d* = 2.8 Å)^75^ and ∼41° (*d* = 2.2 Å)^76^ interplanar distances have been also recently observed and attributed to the in-plane periodicity and semicrystalline structure at the mesoscale. These interpretations give some understanding to the periodicities observed; however, it is clear that the textured growth of the film is strongly related to the air/water interface, as shown in Figure 3c. This is because the interface is the most likely responsible for the highly textured ordering of the films (∼30°), in agreement with some of the models proposed in the literature, but not observed.^54,57^ However, the grains size of the 2D-like membranes and the lack of visible grain boundaries in both SEM and TEM experiments suggest a possible mixture of grains with amorphous PDA parts, while retaining the observed 2D-layered structure.

Nevertheless, a few questions are still open for research, especially since the nanoclusters of PDA have been reported to be highly polycrystalline,^35,66,74^ without any preferential texture, which contradicts the texture observed here. An explanation could be that they are formed by using the preordered stacked molecules at the air/water interface as a “seed” (Figure 3c). This could be supported by the particle inclusions observed in the films, which seem to crack the membranes, suggesting that some spheres are embedded into the membranes while the ones growing on top are fractured by the clusters (see Figure S2a). However, these aspects need to be studied in detail in the future as well as the optimization and control over the membrane/film thickness, optical absorption, and grain size. Further studies are in progress to understand these aspects and the open questions proposed by this study.

## 4 Conclusions

We have prepared free-standing and easily transferable PDA films by a simple method that exploits the air/water interface. The films showed a previously unknown macromolecular arrangement of PDA resembling that of eumelanin clusters. More interestingly, the membranes show a unique layered structure and organization, which resembles that of 2D materials, making them an excellent candidate for further research and integration in several fields. Here, we outline some of the possible applications of our materials. One of the clear candidates is chromatic control and coating technology,^5,77−80^ profiting from the easy transferability of the layers and rather unique structure. The 2D PDA layer could show different optical constants when compared with traditional PDA due to confinement. Moreover, the continuous interest of 2D stacked metamaterials and heterostructures,^81,82^ in which layers from different materials are sandwiched together, could benefit from the inclusion of PDA 2D layers. Also, PDA nanometric coatings have shown enhanced chemical stability toward corrosion^83^ and more recently enhancement of photocatalytic activity when in conjunction with semiconductors,^9^ which could be enhanced by current van der Waals 2D materials. Additionally, here we open the possibility for easy integration in many known architectures in fields such as biomedical applications in wound dressing and antibacterial surfaces of PDA.^7^ Furthermore, the mechanical strength of the membranes is comparable to that of other resilient PDA membranes reported in the literature, allowing many applications on free-standing architectures, such as resonators with a potentially high-quality factor.^28,59^ Finally, our study and films show a set of unique structural and optical properties, which open the possibility for many new studies and experiments in PDA membranes and films.

## Supporting Information Available

Optical images (Figure S1), STEM, SAED, and EDX experiments (Figure S2), EELS thickness analysis (Figure S3), Gi-XRD (Figure S4), XPS for C and O (Figure S5), nanoindentation for thin samples (Figure S6), structural models of PDA (Figure S7), and XPS quantifications (Table S1) (PDF)
