# Supplementary material for: Polydopamine Films with 2D-like Layered Structure and High Mechanical Resilience
Source: ACS Appl Mater Interfaces. 2021 May 10;13(19):23113–20. doi: 10.1021/acsami.1c02483 (PMC8289185; doi:10.1021/acsami.1c02483)
Supplement: Supplementary file 1 — am1c02483_si_001.pdf [file am1c02483_si_001.pdf]

Supporting Information

Polydopamine Films with 2D-Like Layered Structure and High Mechanical Resilience

Emerson Coy[1*], Igor Iatsunskyi[1], Juan Carlos Colmenares[2], Yeonho Kim[3], Radosław Mrówczyński [4*]

[1.] *NanoBioMedical Centre, Adam Mickiewicz University, Wszechnicy Piastowskiej 3, 61-614, Poznan, Poland*
[2.] *Institute of Physical Chemistry, Polish Academy of Sciences, Kasprzaka 44/52, Warsaw, 01-224 Poland*
[3.] *Research Institute of Basic Sciences, Incheon National University, Incheon 22012, Republic of Korea*
[4.] *Faculty of Chemistry, Adam Mickiewicz University, ul. Uniwersytet Poznańskiego 8, 61-614 Poznań, Poland*

Corresponding Authors *: coyeme@amu.edu.pl, radoslaw.mrowczynski@amu.edu.pl

Table of Contents

	Page
**Figure S1:** Optical images of the samples.	**S-2**
**Figure S2:** Scanning electron micrographs, SAED and EDX.	**S-3**
**Figure S3:** EELS thickness studies	**S-4**
**Figure S4:** Gi-XRD for thin PDA film.	**S-5**
**Figure S5:** XPS analysis for C and O regions	**S-7**
**Figure S6:** Nanoindentation of thin membrane	**S-8**
**Figure S7:** PDA structural models	**S-9**
**Table S1:** Components and quantification of XPS spectra	**S-10**

Supporting Information

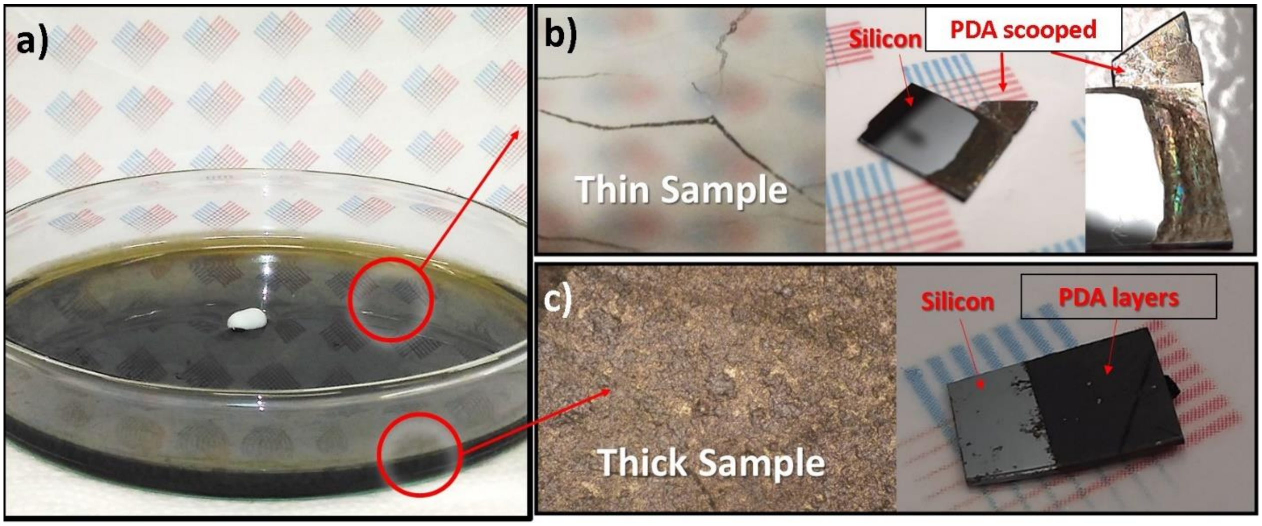

Figure S1: Optical images of the samples collected from the synthesis process. **a)** Shows the collection areas for the samples, as surface and residual after evaporation. **b)** Presents the surface of the dish and the resulting colours and morphology of the scooped sample. **c)** Shows the surface of the resulting thick sample after water evaporation and resulting examined sample.

Supporting Information

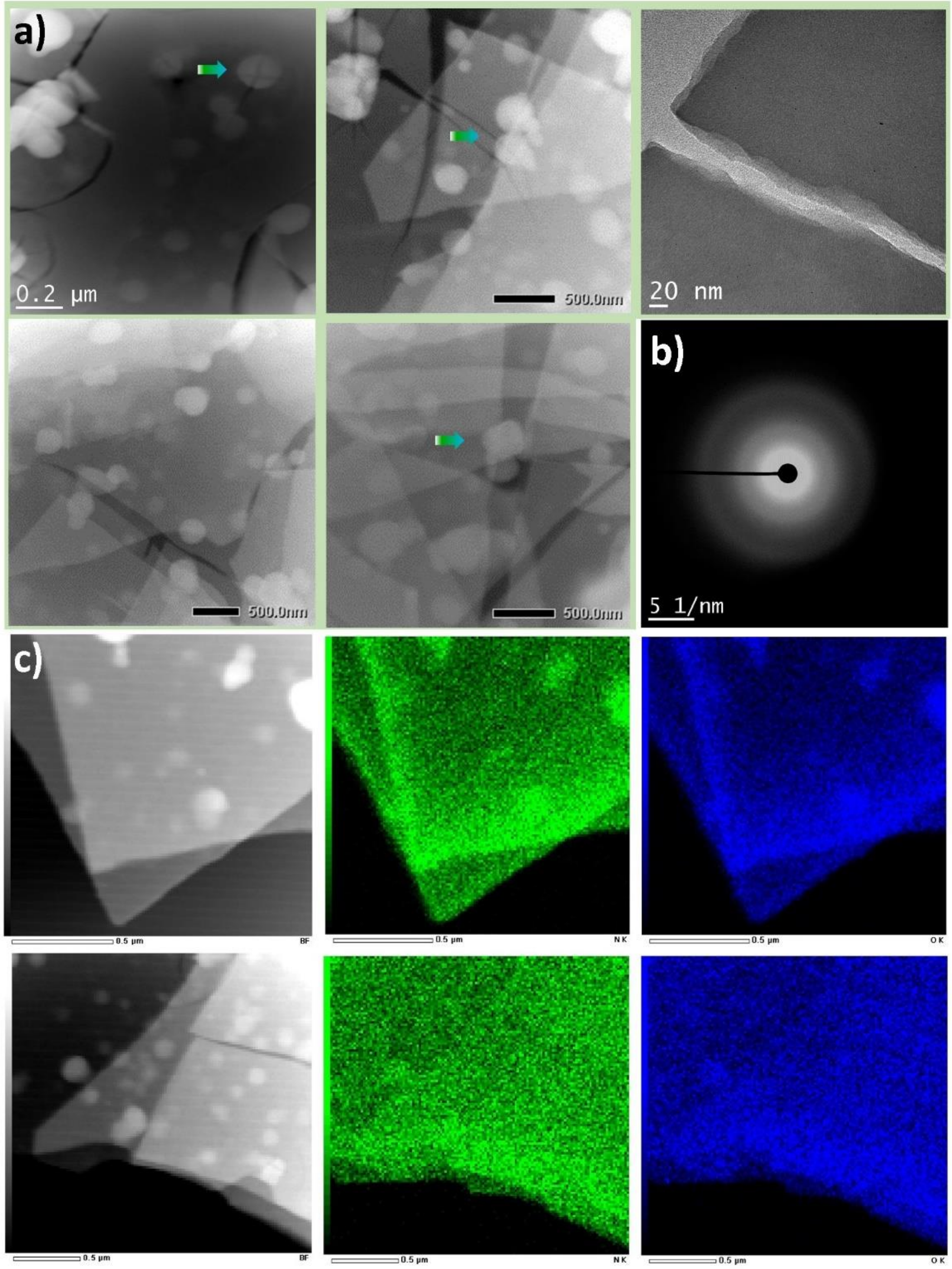

Figure S2: Scanning Micrographs obtained from suspended membranes scooped on Cu grids. a) shows the layered structure of the membranes and the heavily populated particles of different sizes, arrows show places were the particles seem to "crack" the membranes. b) shows the SAED pattern of the membranes with no distinctive morphology. c) EDX measurements performed in the edged section of the suspended membranes mapping N-K and O-K.

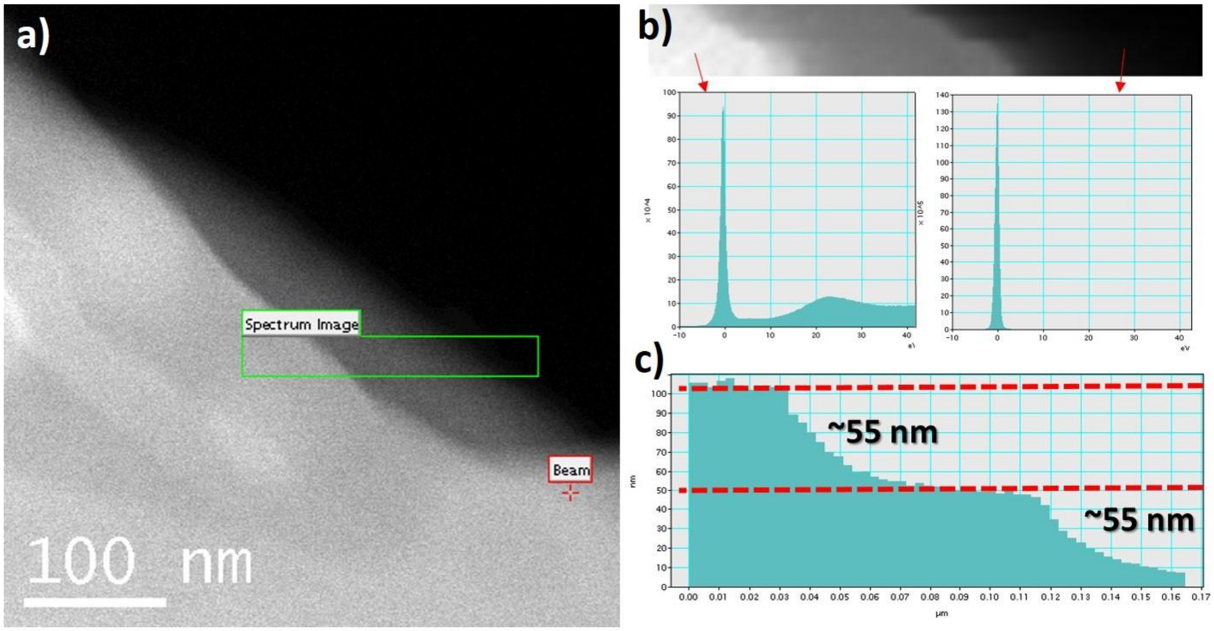

Figure S3: Energy loss spectroscopy (EELS) studies focusing on thickness identification**. a)** Shows the region investigated, **b)** the spectrum image with the EELS spectra for the sample and vacuum section. c) Shows the thickness profile extracted using the Zero loss peak (ZLP) area and the relative intensity relationship $ln(I/Io)$ of the total spectra. The thickness of the layers is around 55 nm.

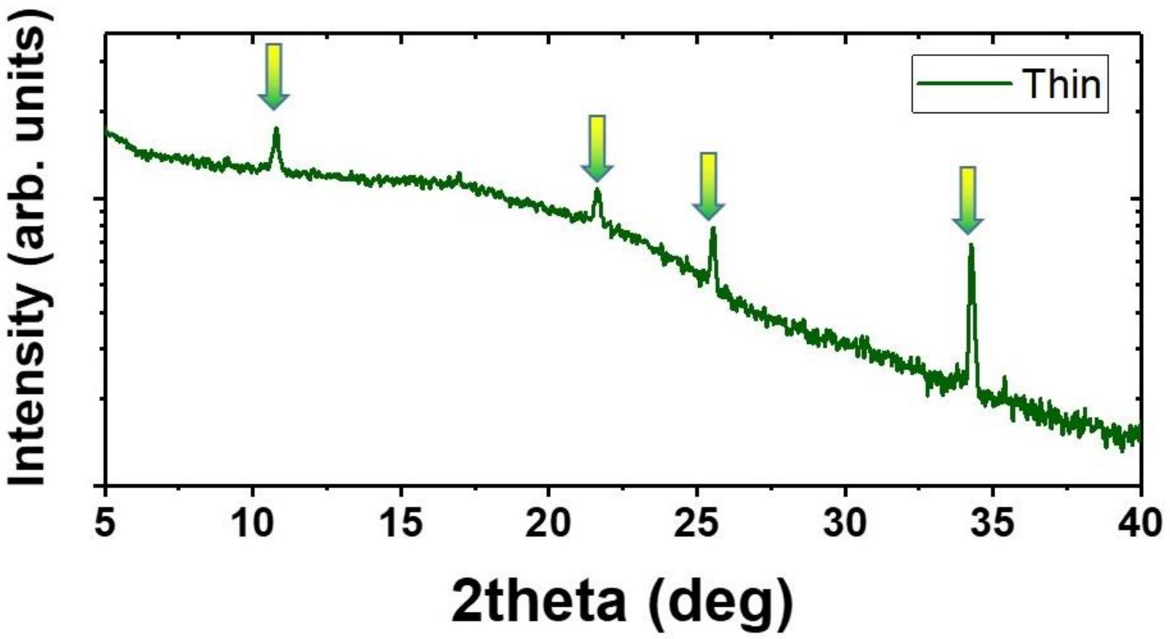

Figure S4: Gracing incident X-ray diffraction experiments performed on the thin sample. The same peaks observed in the XRD experiments for the thick sample are observed, with an additionally broad peak accounting for the amorphous carbon/PDA contribution.

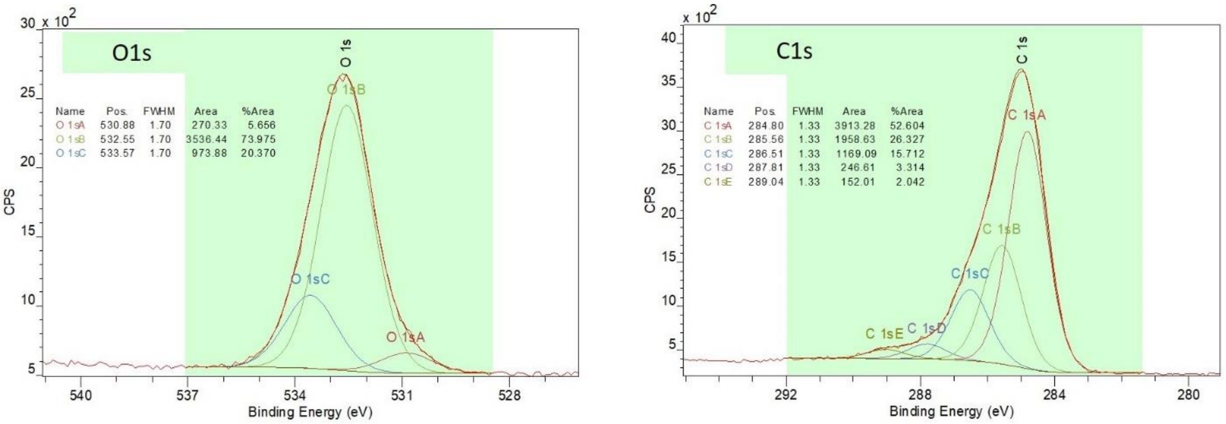

Figure S5: XPS spectra of the PDA thin membrane (left) O1s and (right) C1s with their corresponding fittings and components.

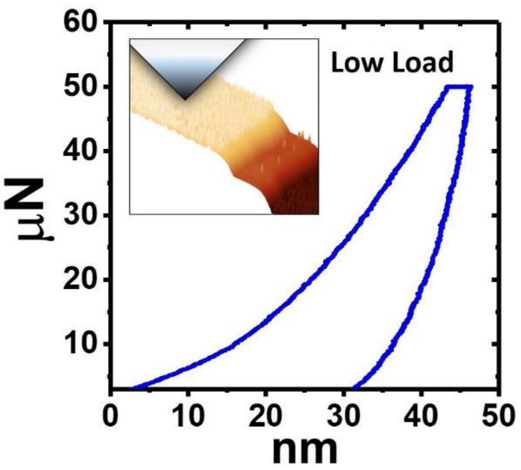

Figure S6: Nanoindentation load vs displacement plot for the thin PDA sample in a bilayer region.

Supporting Information

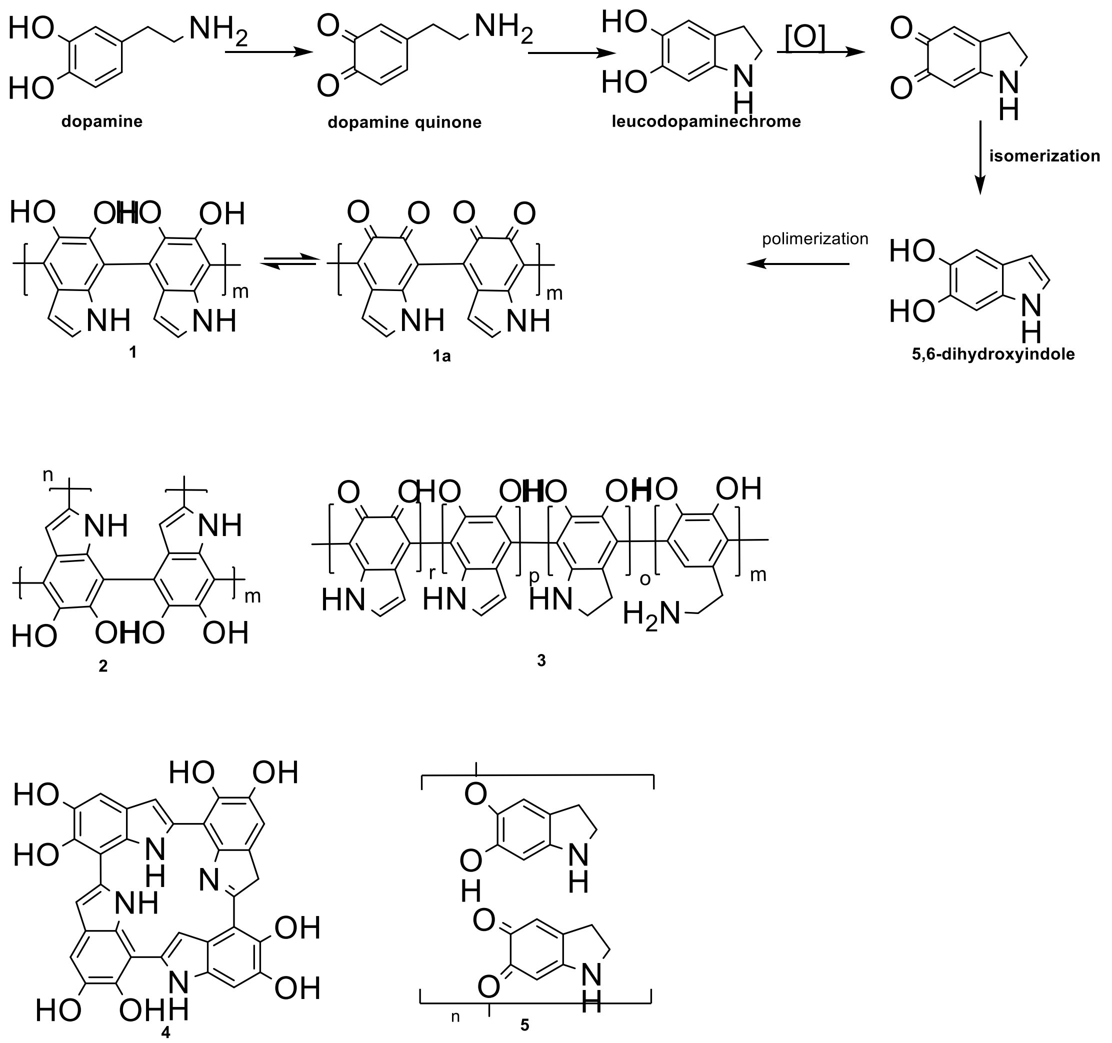

Figure S7: Different structural models for Polydopamine

Supporting Information

Name	Position	FWHM	Library RSF	Raw Area	%At Conc	
O 1sA	530.88	1.7	0.733	270.331	1.15	C=O
O 1sB	532.55	1.7	0.733	3536.44	15.01	C-O
O 1sC	533.57	1.7	0.733	973.878	4.13	SiOx
**O**					**20.29**	
N 1sA	400.05	2	0.499	483.352	3.05	C-N
N 1sB	401.88	2	0.499	175.801	1.11	N(+)
**N**					**4.16**	
C 1sA	284.8	1.33	0.314	3913.28	39.74	C-C
C 1sB	285.56	1.33	0.314	1958.63	19.89	C-N
C 1sC	286.51	1.33	0.314	1169.09	11.87	C-O
C 1sD	287.81	1.33	0.314	246.614	2.50	C=O
C 1sE	289.04	1.33	0.314	152.013	1.54	O=C-O
**C**					**75.55**	

Table S1: Elemental composition of the PDA thin membrane determined by XPS. Oxygen, Nitrogen and Carbon quantification and components.
